# P-950. Pre-Exposure Prophylaxis Education for Internal Medicine Residents (PRIMER)

**DOI:** 10.1093/ofid/ofae631.1140

**Published:** 2025-01-29

**Authors:** Brenton Nash, Jessica Abrantes-Figueiredo, Casey Godshall

**Affiliations:** University of Connecticut, Bloomfield, Connecticut; Saint Francis Hospital, Hartford, Connecticut; UConn Health, Farmington, Connecticut

## Abstract

**Background:**

Pre-exposure prophylaxis (PrEP) for HIV is a safe and effective strategy for HIV prevention among high-risk patient groups. Despite consistent experimental and real-world data showing benefit, PrEP is underutilized. There is a dearth of non-specialized providers engaging in PrEP services. The purpose of this project is to increase knowledge and prescribing of PrEP among a cohort of internal medicine (IM) residents.

**Methods:**

A 3 hour interactive education session was provided to the IM residents of the University of Connecticut Categorical Internal Medicine Residency. Baseline knowledge was assessed with a pre-training survey. This survey was repeated up within six months to assess for a change in PrEP knowledge and prescribing.

**Results:**

The pre-intervention (pre) survey was completed by 39 IM residents. The post-intervention (post) survey was completed by 25 IM residents from the same cohort. 4 participants from the post group were excluded as they had not completed a pre survey. Similar proportions indicated PrEP awareness pre- and post-intervention 97.4% and 100%, respectively. There was a numeric increase in the proportion of respondents reporting knowledge of PrEP indications from 69.2% pre to 85.7% post. Similar proportions reported prescribing PrEP 15.4% pre and 14.3% post. Those who had prescribed PrEP reported providing it to only 1-5 patients. When assessing for paired differences among a sub-set of participants who had both pre- and post scores, general knowledge of PrEP significantly improved. General knowledge scores averaged 8.6% pre and 22.6% post (p-value 0.001). Of the five general knowledge questions – testing individual domains -- the mean differences between pre and post scores were: 0.6 (95% CI 0.3-1.0), 0.2 (0.04-0.4), 0.05 (-0.1-0.2), 0.2 (0.007-0.4), 0.05 (-0.05-0.1), and 0.3 (0.08-0.5), respectively. Indicating that in all but 2 domains the 95% confidence interval did not include 0.

**Conclusion:**

Among a cohort of IM residents from a mid-sized, university-based program, general PrEP knowledge was low despite widespread awareness. Knowledge was improved following an educational session; however, prescribing did not increase. Other barriers besides knowledge likely impact the likelihood of generalists, and in particular learners, to prescribe PrEP.

**Disclosures:**

**All Authors**: No reported disclosures

